# Complex Feeding Tracks of the Sessile Herbivorous Insect *Ophiomyia maura* as a Function of the Defense against Insect Parasitoids

**DOI:** 10.1371/journal.pone.0032594

**Published:** 2012-02-29

**Authors:** Yoshiko Ayabe, Takatoshi Ueno

**Affiliations:** 1 Division of Theoretical Biology, National Institute for Basic Biology, Okazaki, Aichi, Japan; 2 Institute of Biological Control, Faculty of Agriculture, Kyushu University, Fukuoka, Japan; Netherlands Institute of Ecology, Netherlands

## Abstract

Because insect herbivores generally suffer from high mortality due to their natural enemies, reducing the risk of being located by natural enemies is of critical importance for them, forcing them to develop a variety of defensive measures. Larvae of leaf-mining insects lead a sedentary life inside a leaf and make conspicuous feeding tracks called mines, exposing themselves to the potential risk of parasitism. We investigated the defense strategy of the linear leafminer *Ophiomyia maura* Meigen (Diptera: Agromyzidae), by focusing on its mining patterns. We examined whether the leafminer could reduce the risk of being parasitized (1) by making cross structures in the inner area of a leaf to deter parasitoids from tracking the mines due to complex pathways, and (2) by mining along the edge of a leaf to hinder visually searching parasitoids from finding mined leaves due to effective background matching of the mined leaves among intact leaves. We quantified fractal dimension as mine complexity and area of mine in the inner area of the leaf as interior mine density for each sample mine, and analyzed whether these mine traits affected the susceptibility of *O. maura* to parasitism. Our results have shown that an increase in mine complexity with the development of occupying larvae decreases the probability of being parasitized, while interior mine density has no influence on parasitism. These results suggest that the larval development increases the host defense ability through increasing mine complexity. Thus the feeding pattern of these sessile insects has a defensive function by reducing the risk of parasitism.

## Introduction

Defense is a critical component for prey or host species to survive in antagonistic interactions with natural enemies. Prey species have developed a variety of defenses from indirect ones, such as mimicry and cryptic coloration, to direct ones such as biting or moving away from enemies, and the best defense could be to avoid detection by enemies [1]. Many herbivorous insects, however, leave their feeding tracks, which advertise their presence to their enemies by giving away visual and chemical cues. Such advertisement through feeding tracks can critically affect survival rate of them, especially during less mobile or sedentary developmental stages. In this situation, herbivorous insects may develop feeding behaviours, which are directly involved in the formation of feeding track, combined with defensive function to offset or compensate for the disadvantage of feeding tracks. However, very little attention has been paid to defensive aspects of feeding behaviour against enemies [2]–[6]. In the present study, we examined the feeding behaviour as a defensive strategy, using the sessile stages of herbivorous insects, *i.e.* leafminers.

Leafminers live in plant leaf tissue and make conspicuous feeding tracks called mines that provide a visual cue to insect parasitoids and help them to locate leafminers [7]–[10]. Once a mined leaf is detected by a parasitoid, it lands on the leaf and explores the mine by tracking it to find the occupant miner [4], [11], [12]. The occupant leafminer cannot move away from parasitoid attack because its movement is restricted within its own mine. Consequently, leafminers often suffer high levels of mortality from parasitism [13]–[17]. Thus, a mine, or mining behaviour, is a key component in antagonistic relationships with parasitoids because a mine is a direct target for parasitoids and traits of mine could affect the susceptibility of the occupant miners to parasitism.

Several strategies to offset unfavorable features of mines have been proposed. Mining on the lower leaf surface may reduce the probability of detection by parasitoids [18]. This is supported by the fact that upper-surface mining has not led to significant radiations in the phylogeny of the leaf-mining moth *Phyllonorycter* species [19]. Grouping strategy in which multiple miners reside within a single leaf together can decrease per-capita risk of parasitism in the group, although the detection risk of the group increases with the number of mines within a leaf [10]. Shapes and patterns of mines can also function as a defense [2]–[4], [8], [9], [20]. Blotch mines provide the occupant miner larvae with space enough to move and avoid being stung by parasitoids [3], [8], [21].

A unique role of mines as a defense is proposed for linear mines; the branch and cross structures of linear mines should confuse the mine-tracking parasitoids [2], [22]. The hypothesis of linear leafminer defense is supported by several studies. Kato [2] theoretically predicted that parasitoids were more likely to give up host search when confronting mines with more branch structures under the simple assumption that parasitoids track mines for a fixed distance on a mine. Ayabe et al. [4] observed in the laboratory that female parasitoids searched significantly more time on complex mines with crosses than on simple mines without cross. However, no previous studies have given field evidence of defensive role of complex mining patterns.

**Figure 1:**
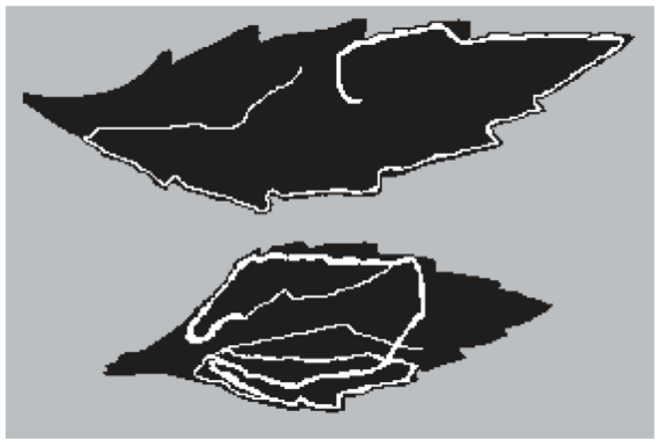
Two example digital images of *Ophiomyia maura* pupal mines. *Ophiomyia maura* frequently feed on at the leaf margin early in its development and the inner part of the lamina later. Mining pattern characteristics: the upper; leaf area = 16.42 cm^2^, total mine area = 1.13 cm^2^, interior mine area = 0.26 cm^2^ and fractal value = 1.13: the lower; leaf area = 9.63 cm^2^, total mine area = 1.67 cm^2^, interior mine area = 1.26 cm^2^ and fractal value = 1.46. The images were digitally scanned and manipulated in color (see the Materials and Methods for details on the manipulation).

We studied the defense of the dipteran leafminer *Ophiomyia maura* Meigen (Diptera: Agromyzidae) that makes white-colored linear mines on the upper-leaf surface of the host plant *Aster microcephalus* (Miq.) Franch. et Savat. var. *ovatus* (Franch. et Savat.). It mines in a characteristic manner: a larva consumes the palisade tissues along the mid vein mining toward the base, then proceeds along the margin of the leaf to the inner area of the leaf during about 4–5 days, and finally moves to the lower side of the leaf to pupate, spending in total about two weeks in the mine [23] (Figure 1). We hypothesized that miner individuals of highly complex mines with more cross structures would be less often parasitized than those of simple mines (the first hypothesis). We also address the ecological meanings of being mined along the margin of a leaf, hypothesizing that mining along the margin of a leaf would reduce the detection risk of the mine and the subsequent parasitism (the second hypothesis); recent studies of crypsis and disruptive coloration have indicated that prey animals can avoid predators' visual detection by obscuring their body outline and a crypsis through background matching [24]–[28]. In bicolored-pattern animals, “monocolor-line” on the edge of their body enhances their body outline, increasing their conspicuousness and hence decreasing their survival rate due to increased predation [24]. However, animals with monocolor-edged pattern can reduce the risk of being detected by lowering density of interior pattern elements to lead to an effective interior background matching [27]. Contextualizing this to the *O. maura*, it is hypothesized that they should face a dilemma of either mining along the margin or mining the interior part of a leaf; although mining along the margin of a leaf may enhance the outline of the mined leaf, it can reduce interior density of mine (the ratio of white mine to green of intact part of the leaf) because the total length of their mine should be constant given they have to feed on a given amount of leaf tissues. Reducing interior density of mine would have effective background matching among other intact leaves. If this is true, *O. maura* individuals of higher interior-density mines are expected to suffer higher parasitism through increased detection risk. Reduced interior density of mine is likely to reduce complexity of mine, which raises tow possibilities; (1) the two defenses through mining patterns are asynchronous, and (2) the two defenses are synchronous and cause intensive parasitism on miner individuals in a certain range of mining pattern traits. Therefore, close examination on the relationship between mining patterns and parasitism would reveal the relative importance of the defense to reduce the detection risk versus the defense to hinder parasitoids' tracking behaviour, *i.e.*, the post-detection defense.

In this paper, we aimed to investigate how mining patterns in the sedentary stages of the leafminer affected the susceptibility to parasitism in the field and to determine the defensive effects among different mining patterns. We used an image analysis to quantify fractal dimensions as complexity of mines and the area of mines in the inner area of the leaf as interior density of mines. These two measurements are concomitantly affected not only with each other but also by the size of the leaf that the mine appears and the length of the mine. Therefore, we used a Principal Component Analysis to control such factors affecting mining pattern traits, which provided accurate evaluation for patterns of mines. Then, we analyzed the relationship between the susceptibility to parasitism and the mining pattern traits with Generalized Linear Models for *O. maura* larvae and pupae. In the models, we also included locations of the leafminer in the habitat as fixed effects since latest studies showed that edge effect caused heterogeneity in parasitism within the habitat [29]–[31]. Finally, we compared parasitism rates between miner individuals situating at two within-leaf positions, *i.e.*, margin versus interior, in order to detect background matching effect of mines at the margin of a leaf.

## Methods

### Site Description

The study site was in the National Institute for Basic Biology, Okazaki City, Aichi Prefecture, Japan (34°57′N, 137°10′W). The site was ca. 8×10 m^2^ and dominated by the deciduous perennial *A. microcephalus*, which is a common species in Japan. The site had no endangered species, and no specific permits were required for the described field studies. In the site, *A. microcephalus* starts growing in late April and blooms in late October. *Ophiomyia maura* occurs from late April to November and has four generations at this site [32]. It occurs solitarily on both leaf and plant scales [32], which precludes the possibility of density-dependent foraging efforts by parasitoids [33]–[38]. Host plants were divided into two groups, according to their density and location in the site (i.e., inner *vs.* outer groups). The plants grew at high density in the inner part of the site (inner group), while at low density in the outer regions.

Approximately 30 mined leaves were randomly collected from each of the two plant groups every two weeks from May through to mid-August 2004, except in late July when the host plants suffered from drought. We could not collect mines after September because plant leaves were seriously damaged due to drought and leafmines were thus too scarce for sampling.

### Leafminer Mortality and the Associated Parasitoid Species

Leaf samples collected were digitally scanned with a scanner (CC-570L, EPSON Co.) and kept in polyethylene bags (4×8 cm^2^) in a room, where air temperatures ranged from 17 to 25°C under natural light. All mines and the occupants were inspected under a microscope without dissection to identify developmental stages (larvae or pupae) and states (live, parasitized, dead from unknown causes, missing, or unknown) of the occupants. When a mine developed into the lower leaf side to make a pupation site, the mine was referred to as a pupal mine, otherwise, as a larval mine. In identifying the state of the occupants, we used a working definition for parasitism (see also the next section for the definition of parasitism). For empty mines, we identified the state with the following rules; we referred to empty pupal mines with an eclosion slit at the pupation site as ‘live’; empty larval and pupal mines with a round hole made by the parasitoid at their emergence as ‘parasitized’; larval mines whose occupants were absent and lacked a piece of leaf tissue as ‘missing’. When we could not identify the state of the occupants, it was defined as ‘unknown’. We compared mortality factors between *O. maura* larvae and pupae by using a chi-squared test. Mortality factors were also compared between larval positions within a leaf (the inner part *vs.* the marginal part of the leaf) to test the hypothesis that larvae mining at the margin were less often parasitized than those in the inner part of the leaf due to the reduced interior density of mine. This analysis was not performed for pupae because all pupae were situated in the inner part of the leaf. The leaves containing parasitoids were kept to identify the species.

### Description of Parasitism

*Ophiomyia maura* can be attacked by both idiobiont and koinobiont parasitoids, but in this study, we focused only on the attack by idiobiont parasitoids. Idiobiont parasitoids kill or permanently paralyze hosts at oviposition [39], and therefore we can obtain information on the mining patterns when the hosts have been attacked. On the other hand, koinobiont parasitoids do not kill hosts at oviposition and allow further host development, and for this reason it is not possible to accurately detect the relationship between mining patterns of *O. maura* and the susceptibility to parasitism. Idiobiont parasitoids are responsible for the majority of leafminer parasitoid assemblages because they are supposed to be superior in competition with koinobiont parasitoids [39]–[41]. Leafminer samples from which koinobiont parasitoids had emerged were classified into “live” as state of leafminers. Parasitoids were identified at species level. In the case that parasitoid species could not be identified due to death before emergence, the type (*i.e.*, idiobiont or koinobiont) of the parasitoids was determined by scrutinizing the development of the mines and the occupant individuals.

### Analyses of the Effects of Mining Patterns on Parasitism

Leaf area can affect the mining pattern through the length of the leaf margin available for a larva, and length of mine can also affect the mining pattern because long mines are more likely to have cross structures than short mines do. Therefore, to evaluate the characteristics of the mining pattern of each sampled mine, we measured (1) the area of the leaf, (2) the total area of the mine, (3) the area of the mine in the inner part of the leaf as the interior density of mine, and (4) fractal dimension as a measure of mine complexity. The mine area was a proxy for mine length because several mines were so highly tortuous that their length could not be measured accurately. Mine area significantly correlated with mine length for 48 simple-patterned pupal mines (regression analysis: mine area [cm^2^] = 0.07×mine length [cm]; *R* ^2^ = 0.92, *N* = 48, *P*<0.001). The scanned images of the sample mines were transformed into digital forms and converted to grayscale using the image analysis software, NIH Image 1.63 (http://rsb.info.nih.gov/nih-image/). Leaf and mine areas were each manually painted with different shades of gray (see Figure 1). We then counted pixels of the same gray shade to evaluate leaf or mine area, where 1 cm^2^ was approximately 840 pixels in our scan system. We subtracted the area of the mine bordering on the edge of the leaf from the whole area of the mine to evaluate the interior density of mine. The complexity of mines is best evaluated with the numbers of cross and branch structures that can mislead parasitoids during tracking on mines [2], [4]. However, several mines are so highly tortuous that we cannot count cross structures accurately. We instead evaluated fractal dimension, which was a description of complexity of a geometric object [42], allowed us to evaluate objectively the complexity of mines as a quantitative character and correlated with the number of cross structures [4]. The fractal value cannot only take integer but also non-integer values; a leafmine can take a fractal value in the range from one to two, and a high fractal value means that the mine is highly complex. We used the box-counting method to estimate fractal dimensions of the scanned mine images (for details, see [42], [43]). Leaf area, total mine area, interior mine area and fractal values were compared between *O. maura* larvae and pupae using two-sample *t* tests.

In a preliminary analysis, we found the multicolinearity of the above four measurements. Therefore, we used a principal component analysis (PCA) to replace the original correlating measurements (variables) by a series of uncorrelated linear combinations of the data, i.e. principal components (PCs). Principal components were extracted from the correlation matrix of the variables, and the underlying structures of the four measurements were deduced. Scores of the newly estimated PCs were used in the Generalized Linear Model (GLM) with the binomial distribution and logit link functions to investigate whether mining patterns could affect the susceptibility of *O. maura* larvae and pupae to parasitism. In the GLMs, we also included the sampling locations within the habitat (i.e., outer vs. inner groups) as an independent dummy variable, with value 1 for the inner group and 0 for the outer group. We did not consider mine density per leaf as an independent variable in the model because *O. maura* solitarily utilizes a leaf on its own [32]. In the PCA and GLM, we used the mines that had been classified as live and parasitized in the assessment of *O. maura* mortality (*n* = 58 and 85 for larval and pupal mines, respectively). With respect to the analysis of larval parasitism, pupae were included as live individuals because pupae had been under the risk of parasitism until just before pupation and could avoid the attack by larval parasitoids successfully. This suggests that surviving individuals may have an obviously different pattern from parasitized ones because surviving larvae continue mining while the parasitized ones stop mining. Under a random sampling process, however, we expect that the rate of parasitism should be constant over the duration of mining by a larva from hatching until just before pupation, unless parasitism is affected by any factor such as the mining patterns.

The colinearity between interior density of mine and complexity may lead to an insignificant result in the GLMs because the influence of one mine pattern trait can be offset by the other. Therefore, we visualized the effects of the mine pattern traits represented with PCs on parasitism of *O. maura* using lowess curves, which provide locally weighed scatter plot smoothing and follow the trends of the data. All statistical analyses were performed using Stata 9 [44].

## Results

### Leafminer Mortality and Parasitoid Assemblage

**Figure 2:**
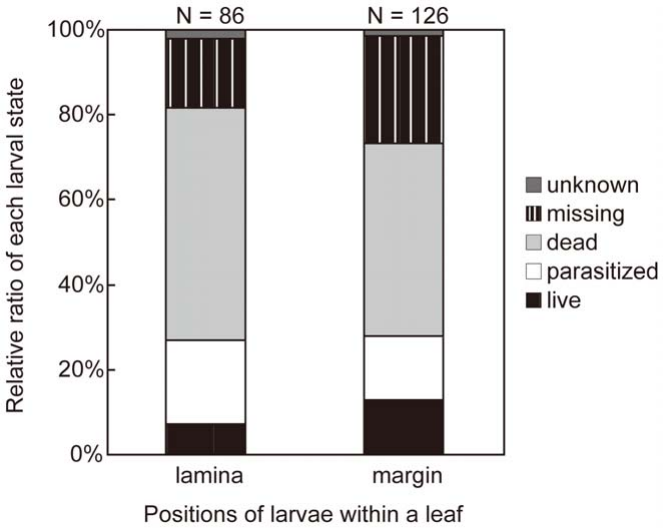
Relative state ratio of *Ophiomyia maura* larvae at the margin/inner part of the leaf.

We collected 212 *O. maura* larvae and 136 pupae in the field. The leafminers collected were classified into 5 states (i.e., live, parasitized, dead from unknown cuases, missing, unknown), and the composition of host states differed significantly between larval and pupal stages (Chi-square test: *χ* ^2^_4_ = 122.9, *N* = 348, *P*<0.0001, Table 1). The host condition observed most frequently was death from unknown causes in the larval stage (49.1%), while in the pupal stage it was parasitism (33.1%). Among the 348 mines collected, 17.8% of the occupants were alive, while 23.3% were parasitized. Host states did not significantly differ between larvae in the inner part and those at the margin of the leaf (Fisher's exact probability test: *χ* ^2^_4_ = 5.30, *N* = 212, *P* = 0.244, Figure 2). This suggested that miner individuals mining at the margin, which had lower interior pattern density, obtained no advantage in avoidance of parasitism.

**Table 1:** *O. maura* larval and pupal states.

	States					
*O. maura* stage	Live*	Parasitized	Dead	Missing	Unknown	Subtotal
Larvae (%)	22 (10.4)	36 (17.0)	104 (49.1)	46 (21.6)	4 (1.9)	212 (100)
Pupae (%)	40 (29.4)	45 (33.1)	2 (1.5)	36 (26.5)	13 (9.5)	136 (100)
Subtotal (%)	62 (17.8)	81 (23.3)	106 (30.4)	82 (23.6)	17 (4.9)	348 (100)

* Leafminers attacked by koinobiont parasitoids were included in “live” (*N* = 7).

We collected three idiobiont and two koinobiont parasitoid species from *O. maura* mines, and idiobiont species accounted for over 90% of the collected parasitoids (Table 2). The dominant species was the pupal idiobiont parasitoid *Pediobius metallicus*, followed by the larval idiobiont parasitoid *Chrysocaris pentheus*.

**Table 2:** The parasitoids associated with *Ophiomyia maura*.

Type	Parasitoid species	Host stage	N	Frequency
Idiobiont	*Chrysocharis pentheus* (Waker) (Eulophidae)	Larva	16	0.19
	*Pediobius metallicus* (Nees) (Eulophidae)	Pupa	31	0.38
	*Sphegigaster hamugurivora* Ishii (Pteromalidae)	Pupa	3	0.04
	Unknown*	Pupa/Larva	31	0.39
Koinobiont	*Dacnusa nipponica* Takada (Braconidae)	Larva	1	0.29
	*Opius* sp. (Braconidae)	Larva	2	0.14
	Unknown*	Pupa/Larva	4	0.57

* Parasitoid species was not identified because the parasitoid had already emerged or because the parasitoid was dead before emergence.

### Analyses of Mining Patterns

Average leaf area ± SE was 11.93±0.34 cm^2^ for leaves with larval mines (*N* = 212) and 11.79±0.42 cm^2^ for leaves with pupal mines (*N* = 135), and there was no significant difference between them (Two sample *t* test: *t*_345_ = 0.255, *P* = 0.799). Average size ± SE of larval mines (0.54±0.03 cm^2^, *N* = 212) was significantly smaller than that of pupal mines (1.50±0.03 cm^2^, *N* = 135) (Two sample *t* test: *t*_345_ = −22.46, *P*<0.0001). The interior density of larval mines (0.36±0.03 cm^2^, *N* = 58) was smaller than that of pupal mines (1.02±0.04 cm^2^, *N* = 85) (Two sample *t* test: *t*_141_ = −11.02, *P*<0.0001). Approximately 35% of the whole size of the pupal mines fitted along the leaf margin. The mean fractal value of larval mines (1.11±0.008 [± SE], *N* = 212) was also smaller than that of pupal mines (1.29±0.01, *N* = 135) (Two sample *t* test: *t*_345_ = −15.12, *P*<0.0001).

The results of the PCA are summarized in Table 3. In both larval and pupal mines, the first two components explained approximately 90% of the total variation. The first principal components (PC1) on both larval and pupal mines were interpreted as the mining pattern; a higher score meant that the mine was larger ( = longer and well developed), higher interior mine density, and more complex, while a lower (negative) PC1 score meant the opposite. With PC1 scores, we therefore were able to determine how interior mine density and mine complexity are involved in parasitoid attack in the following GLM analyses and Lowess curves. PC2 on both larval and pupal mines mainly reflected the variation in leaf area, with higher scores meaning that mines were made on larger leaves.

**Table 3:** Variable eigenvectors in principal components for *Ophiomyia maura* mines.

Mines	Variables	PC1	PC2	PC3	PC4
Larval mines (N = 143)*	Leaf area	−0.189	0.905	0.234	0.301
	Total mine area	0.581	0.189	0.513	−0.603
	Interior mine area	0.514	0.334	−0.787	0.069
	Fractal value	0.602	−0.184	0.250	0.736
	Variation (%)†	60.41	26.93	9.76	2.91
	Cumulative (%)	60.41	87.33	97.09	100
Pupal mines (N = 85)	Leaf area	−0.329	0.758	0.412	0.384
	Total mine area	0.499	0.485	−0.685	0.209
	Interior mine area	0.565	0.317	0.445	−0.619
	Fractal value	0.570	−0.301	0.399	0.653
	Variation (%)†	62.57	29.52	5.03	2.88
	Cumulative (%)	62.57	92.09	97.12	100

* The data of pupal mines were also included (see text for the reason).

† Variation explained by each PC.

### Effects of Mine Patterns on Parasitism

**Figure 3:**
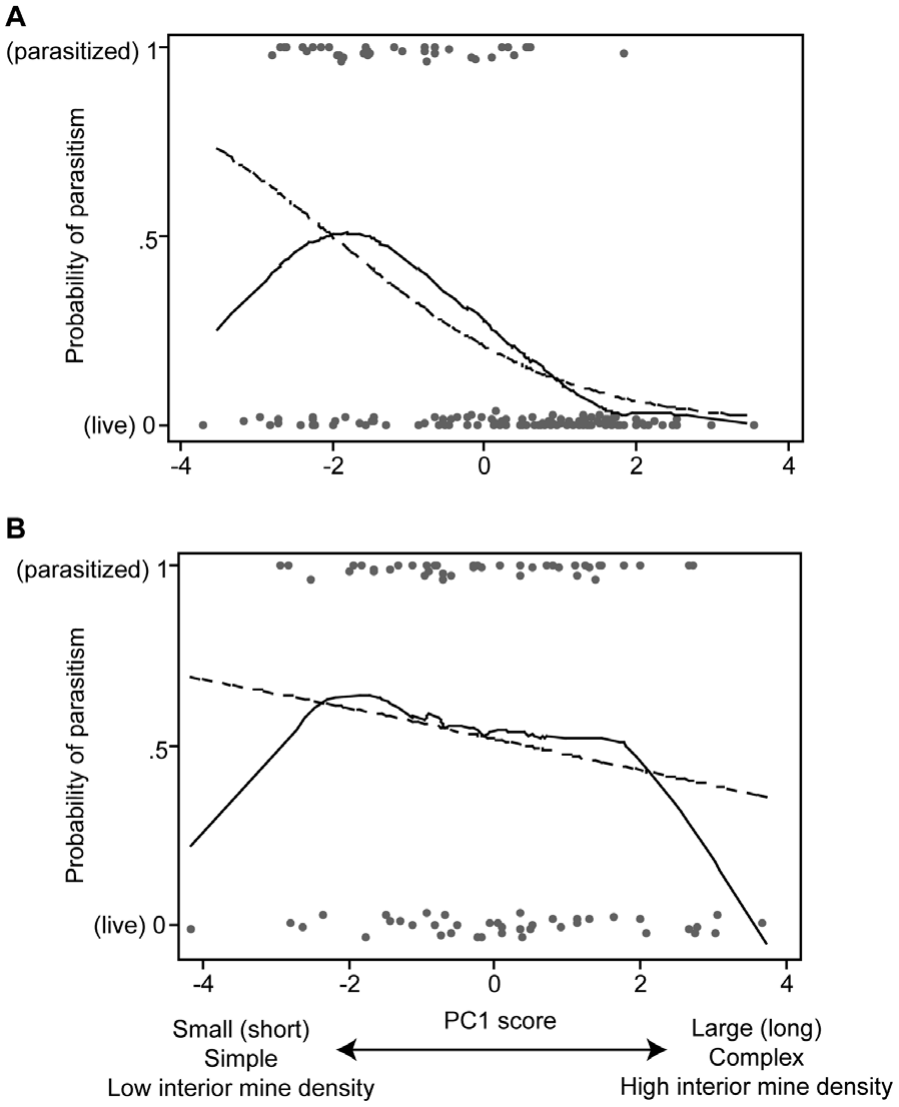
The relationships of *Opiomyia maura* (a) larval and (b) pupal parasitism with mining pattern traits. Mining pattern traits are represented by PC1 scores. A high PC1 score means a long (well developed), high interior-density and highly complex mine. Solid and dashed lines indicate lowess and logistic curves, respectively.

The parasitism of *O. mara* larvae was significantly affected by PC1 score (Table 4; GLM: AIC = 0.98, BIC = −558.27, LR *χ* ^2^_3_ = 29.79, *N* = 143, *P*<0.0001); the negative coefficient of PC1 meant that parasitism occurred less often on larvae of which mines were of longer, more complex, and higher interior mine density. Thus, higher complex mining patterns reduced parasitism, which was consistent with our first hypothesis, while higher interior mine density did not increase parasitism, which was opposite to our second hypothesis. PC2 also had a significant effect on larval parasitism, indicating that parasitism occurred more frequently on larvae inside smaller leaves. Locations at which larvae were found within the habitat did not affect the larval parasitism. The lowess curve of the larval parasitism was convex upward, with a peak at a small negative PC1 score (Figure 3A). Although showing a trend different from that of the logistic curve in the range of PC1 score from −4 to −2, both analyses reached the same conclusion that *O. maura* larvae of moderately short, low interior-density and simple mines were most susceptible to parasitism.

**Table 4:** Effects of sampling designs and mining patterns on *Ophiomyia maura* larval and pupal parasitism*.

Mines	Sources†	Coefficinet	Standard coefficient‡	Z	P
Larval mines	Sampling groups	0.42	0.21	0.94	0.345
	PC1	−0.69	−1.07	−4.55	<0.0001
	PC2	−0.44	−0.46	−1.98	0.048
Pupal mines	Sampling groups	−0.014	−0.007	−0.03	0.975
	PC1	−0.18	−0.28	−1.24	0.215
	PC2	−0.22	−0.24	−1.07	0.286

* Parasitism was treated as a dummy variable; it took 1 if a leafminer was parasitized, or 0 otherwise.

† Sampling group was a dummy variable that took 1 for leafminers collected from the inner group, or 0 for leafminers collected from the outer group.

‡ Standardized coefficients were calculated to compare the relative contribution of each independent variable.

In pupal mines, none of the factors affected parasitism (Table 4; GLM: AIC = 1.45, BIC = −244.87, LR *χ* ^2^_3_ = 2.74, *N* = 85, *P* = 0.43). The lowess curve of pupae was flat over the whole range of PC1 score (Figure 3B). Thus, Mining patterns of *O. maura* pupae were not involved in the susceptibility of them to parasitism.

## Discussion

Our hypothesis was that *O. maura* individuals inside highly complex and/or low interior-density mines suffered less often parasitism because such mine traits would reduce the attack risk of the occupants and/or the detection risk of the mines by parasitoids. Our results showed that larval parasitism decreased with increasing complexity and interior density of mines. Hence our hypothesis was supported with respect to defense by complex mining patterns (the first hypothesis) but not to defense by mining along the margin of a leaf (the second hypothesis). Thus, the feeding track of the sessile herbivorous insect was demonstrated to have defensive consequences against parasitoids attack through the complex pattern.

In general, idiobiont parasitoids prefer large hosts that can provide high fitness return [39], [45]. If this is the case with idiobiont parasitoids of *O. maura*, the dominant larval parasitoid *C. pentheus* should have concentrated on large host larvae that are expected to be found inside long mines with a complex pattern. Furthermore, larger larvae have been exposed to the risk of parasitism for longer period of time. However, the results of GLM and the lowess curve against larval PC1 scores showed that parasitism was actually concentrated among the small larvae (Figure 3A), reinforcing the defensive role against parasitoids of highly complex pattern in mines. This also means that host defense through complex mining patterns constrains host size selection by idiobiont parasitoids and may cause a parasitoid population consisting of small-sized individuals with low fecundity. Ayabe et al. [4] showed that highly complex mines of the dipteran serpentine leafminer *Liriomyza trifolii* increased search time of parasitoids on them, in comparison with simple mines because encountering a crossing in the mine forced the parasitoids to repeat attempts of mine tracking. A similar mechanism is likely involved in the present system.

Intensive parasitism among host individuals inside small mines has also been reported for the lepidopteran blotch leafminer *Antispila nysaefoliella* [9]. This may be because larvae inside larger mines produce more intense or confusing vibrations and are more effective in evading a parasitoid searching from the leaf surface via vibrational sounding and antennation [46]. A parasitoid species of the serpentine leafminer *L. trifolii* also exhibits vibration-related mine-tracking behaviour, and it is suggested that tortuous and crossing patterns of mines confuse tracking of mines by parasitoids [12]. Vibration is a key component in leafminer-parasitoid systems and affects both the defense by leafminers and the foraging behaviour of parasitoids [21], [47]–[50]. Regardless of the type of mine (blotch or linear mines), the enlargement of mines during development of the occupant larvae could decrease the susceptibility to parasitism through confused vibration. In addition, it must be noted that there is another possibility to explain the highest parasitism for small larvae. The larval feeding rate, *i.e.*, the rate of mine development, increases as the occupant larvae grow [51], which may decrease the probability of being attacked per unit length of mine. If this could be the case, the parasitism level would be lowered for large larvae.

Unlike larval parasitism, pupal parasitism was not affected by mine complexity. Lack of defense by occupants of pupal mines may explain the high rate of parasitism in pupae (Table 1). Compared to the larval mine patterns, which vary from short and simple just after hatching to long and complex just before pupation, the variation in pupal mine patterns seems to be too small to affect parasitoid foraging and rate of parasitism. For another possible explanation, pupal parasitoids may have a foraging strategy different from that of larval parasitoids. Pupal parasitoids may directly locate host pupae from the lower leaf surface because *O. maura* pupate on the lower-side of the leaf; tracking of the upper-surface mines may be an ineffective strategy for pupal parasitoids. A high level of pupal parasitism (Table 1) suggests that the pupal parasitoids of *O. maura* can adapt to the mining position change by *O. maura*, although pupation at lower leaf surface would eliminate pupal parasitoids species that searches on the upper leaf surface exclusively.

In *O. maura*, we could not detect any advantage of mining along the margin of a leaf in reducing parasitism during the larval and pupal stages. In addition, locations of larval individuals within a leaf (the inner vs. the marginal part of the leaf) did not affect the susceptibility of leafminers to parasitism. Therefore, our field investigation implies that mining along the margin of a leaf and/or reducing interior mine density are not effective in background matching of the mined leaf among intact leaves. Parasitoids would use odorous and acoustic cues associated with host miners as well as visual cue of mines to locate leafminers [7]–[10]. Further experiments on the behavioural response of parasitoids to a variety of mines with different interior mine density and to a variety of outline patterns of mines will provide fascinating insights into the importance of visual cue in leafminer parasitoids' foraging.

Mining along the margin of a leaf in *O. maura* can be explained from a perspective of nutritional variability of a leaf. *Ophiomyia maura* larvae can obtain higher nitrogen content from the leaf tissues at the margin than in the inner part of the leaf [23]. In addition, the arrangement of the midrib and lateral veins of the host plant (i.e., pinnate venation) has an influence on the mining pattern of *O. maura*. Feeding on the leaf margin enables *O. maura* larvae to avoid the midribs and the lateral veins with well-developed parenchyma cells, which is not eaten by *O. maura* larvae [23]. In other agromyzid leafminers, mining patterns are linked with the selective feeding by the occupant larvae for the tissues that are rich in nutritional contents and/or poor in structural and chemical defenses [52]–[54].

The result of the GLM showed that the larval parasitism had a positive relationship with PC2, suggesting higher parasitism for larvae inside smaller leaves. Small leaf area means that such leaves have emerged relatively recently at the apical growth point and are positioned in the upper part of the plant. *Ophiomyia maura* preferentially utilizes newly emerged leaves in the upper position within a plant for oviposition [32]. Hence, the significant effect of PC1 and PC2 on parasitism implies that parasitoids preferentially attack small, early instars of *O. maura* that are present in small leaves at the upper position within a plant. Large and later instars inside complex mines on leaves at the lower position would be not parasitized due to their complex mines, and for another reason, they would be of advantage through the covering effect by the upper young leaves of the same plant individual.

The insignificant effect of pupal PC1 score means that *O. maura* mining pattern traits are unlikely to be selected as adaptive defensive traits against parasitoids because variation in pupal mine patterns might be due to the stochastic phenotype variation among individuals. In contrast, variation of larval mine patterns can strongly reflect the variation in the degree of larval development. Therefore, the defensive effect of complex patterns in *O. maura* larval mines may be concomitantly brought about by larval development. The fact that *O. maura* juvenile larvae early in development are more susceptible to parasitism implies that *O. maura* individuals that grow fast will be favoured by natural selection.

In the present study, we took account of possible effects affecting the risk of parasitism, *i.e.*, edge effects, to address directly the issue of leafminer defense with mining patterns under field conditions. Edge effect was not detected, and our study hence offers host defense as a contributing factor to heterogeneity in parasitism in host population in the field. Recently, a growing number of studies have focused on the defense of leafminers against parasitoids in relation to mine characteristics, such as color, shape, and pattern [2]–[4], [9], [10], [20]. Some leafminer species change their feeding site during mining from the upper side to lower side of the leaf, and *vice versa*, which may function to avoid parasitism. Further study will provide more profound insights into the adaptive significance and the evolution of mining behaviour, by investigating the relationships among the level of parasitism, the development time while residing inside mines (i.e., the level of exposure to parasitism), and mine characteristics among various leafminer species.
